# Plant-derived smoke water and karrikinolide (KAR_1_) enhance physiological activities, essential oil yield and bioactive constituents of *Mentha arvensis* L.

**DOI:** 10.3389/fpls.2023.1129130

**Published:** 2023-04-20

**Authors:** Sarika Singh, Moin Uddin, Aman Sobia Chishti, Urooj Hassan Bhat, Sangram Singh, M. Masroor A. Khan

**Affiliations:** ^1^ Plant Physiology Section, Department of Botany, Aligarh Muslim University, Aligarh, India; ^2^ Botany Section, Women’s College, Aligarh Muslim University, Aligarh, India

**Keywords:** plant-derived smoke water, karrikinolide, essential oil yield, bioactive constituents, *Mentha arvensis* L.

## Abstract

**Introduction:**

The current study was carried out with the hypothesis that foliar application of plant-derived smoke water (PDSW) and karrikinolide (KAR_1_) might enhanced the plant growth, physiology, and essential oil production of the *Mentha arvensis* L. Karrikinolide (KAR_1_) is one of the most important bioactive constituents of PDSW.

**Methods:**

Mint (*Mentha arvensis* L.) was grown in natural conditions in the net-house. Different concentrations of PDSW (1:125, 1:250, 1:500 and 1:1000 v/v) and KAR_1_ (10^-9^ M, 10^-8^ M, 10^-7^ M and 10^-6^ M) were used as foliar-spray treatments, using double-distilled water as control. The PDSW was prepared by burning the dried wheat-straw that acted as a growth-promoting substance.

**Results:**

Foliar-spray treatment 1:500 v/v of PDSW and 10^-8^ M of KAR_1_ proved optimal for enhancing all morphological, physiological, and essential-oil yield related parameters. In comparison with the control, 1:500 v/v of PDSW and 10^-8^ M of KAR_1_ increased significantly (*p ≤ 0.05*) the height of mint plant (19.23% and 16.47%), fresh weight (19.30% and 17.44%), dry weight (35.36% and 24.75%), leaf area (18.22% and 17.46%), and leaf yield per plant (28.41% and 23.74%). In addition, these treatments also significantly increased the photosynthetic parameters, including chlorophyll fluorescence (12.10% and 11.41%), total chlorophyll content (25.70% and 20.77%), and total carotenoid content (29.77% and 27.18%). Likewise, 1:500 v/v of PDSW and 10^-8^ M of KAR_1_ significantly increased the essential-oil content (37.09% and 32.25%), essential oil productivity per plant (72.22% and 66.66%), menthol content (29.94% and 25.42%), menthyl acetate content (36.90% and 31.73%), and menthone content (44.38% and 37.75%). Furthermore, the TIC chromatogram of the GCMS analysis revealed the presence of 34 compounds, 12 of which showed major peak areas.

**Discussion:**

Treatment 1: 500 v/v of PDSW proved better than the treatment 10^-8^ M of KAR_1_ with regard to most of the parameters studied. The outcome of the study can be used as a recommendation tool for agricultural and horticultural crops, since it costs much lesser than that of KAR_1_. In fact, the foliar application of PDSW proved economical and played bioactive role at very low concentrations.

## Introduction

Medicinal and aromatic plants (MAPs) have played a significant role in human healthcare. Products of MAPs comprise valuable components linked to human healthcare systems in addition to contributing significantly to the cosmetic and industrial sectors of developing countries (Gómez-Galera et al., 2007). Extraction of natural substances from MAPs has been used by our forefathers in the curing of illness, wounds, and suffering for hundreds of years. In the current scenario, almost 80% of the world population, from both developed and developing countries, is still dependent on the MAPs for healthcare needs (Máthé, 2015). According to a report by Kamboj (2000), as many as 20,000 species of medicinal plants have been recorded, out of which nearly 800 species have been used as phytochemical agents for curing various diseases.

*Mentha arvensis* L., commonly called field mint, corn mint, or *pudina*, is a medicinally important crop. The Japanese plant *Mentha arvensis* L., also known as “mint,” is a member of the Lamiaceae family and is widely grown in temperate areas of Europe, western and central Asia, and North America (Aflatuni et al., 2005). Essential oil of mint plant contains several secondary metabolites including menthol, menthone, menthyl acetate, neomenthol, α-pinene, β-pinene, piperitone, limonene, tannins, flavonoids (Akram et al., 2011). In addition to possessing pharmacological properties, the extracts of mint leaves have been shown to carry analgesic, antiseptic, antispasmodic, antifungal, and antibacterial activities (Akram et al., 2011). Menthol, derived from the essential oil (EO) of the mint plant, contains medicinal properties against skin allergies; it is an antiseptic and carminative agent, having diuretic properties as well. (Thawkar, 2016).

In post-fire landscapes, smoke acts as an important abiotic factor that helps plant regeneration. Several studies supported the idea that pyrolysis products derived from burned plant material could be used to stimulate seed germination (Nelson et al., 2012). Flematti et al. (2004) isolated and purified 3-methyl-2H-furo [2, 3-c] pyran-2-one from plant-derived smoke as a bioactive constituent. Following that, several karrikins analogs were isolated and named after 3-methyl-2H-furo [2, 3-c] pyran-2-one (Flematti et al., 2007; Dixon et al., 2009).The chemical structure of karrikins is characterized by a methyl-butenolide moiety, which shares structural similarities with strigolactone, a plant-synthesized endogenous hormone first identified as a germination stimulant in seeds of parasitic Orobanchaceae species (Waters and Smith, 2013). Since Akeel et al. (2019) found that plant-derived smoke water (PDSW) and karrikin (KAR_1_) are the best stimulants for seed germination and different plant characteristics, the goal of this study was to find out how foliar application of PDSW and KAR1 affects the mint plant in terms of growth characteristics, physiological characteristics, and parameters associated to the essential oil.

## Materials and methods

### Plant material and treatments

The experiments was performed on the mint plant (*Mentha arvensis* L.), variety *Kosi* in the net house of Botany department, AMU, Aligarh. Suckers of mint were purchased from Central Institute of Medicinal and Aromatic Plants, Pantnagar, Uttarakhand, India. Uniform suckers of mint plant were surface sterilised with 5% NaOCl solution for 5 minutes, followed by repetitive washing with double distilled water. Suckers were sown in earthen pots (25 ×25 cm) having 5 kg of soil mixture including manure in 6:1 ratio. Plant-derived smoke water was prepared using a laboratory set-up in which burning fumes of dried wheat straw were passed into a suction flask containing 1 L of DDW (double-distilled water) through a continuous bubbling procedure (Akeel et al., 2019). Five samples of PDSW was taken in 5 different test tubes, and the OD (optical density) of the PDSW was noted every five minutes using a spectrophotometer (Shimadzu UV-1700, Tokyo, Japan) until a smoke saturation level was reached, showing no further increase in the OD of the smoke water. Thereafter, the smoke solution was strained through Whatman filter paper No. 1, considering it a stock solution for further dilutions. Four different dilutions of plant derived smoke water (1:125, 1:250, 1:500, and 1:1000 v/v) were prepared using DDW. For preparation of 1:125 v/v PDSW solution, add 8 mL of stock-solution to 992 mL of DDW. Likewise for 1:250 v/v solution, add 4 mL of PDSW stock solution to 996 mL of DDW, for 1:500 v/v concentration, add 2mL of PDSW stock solution to 998 mL of DDW and for 1:1000 v/v concentration, add 1mL of PDSW stock solution to 999 mL of DDW. Control plants were sprayed with DDW only. Foliar application of PDSW treatments was carried out five times within an interval of 5 days between two treatments.

Similar experiment was performed on mint plant with different dilutions of karrikinolide (KAR**_1_**), viz., 10^-9^ M, 10^-8^ M, 10^-7^ M and 10^-6^ M, through a foliar-feeding procedure. 1mg of KAR_1_ is dissolved in 666 mL of DDW to prepare 10^-5^ M stock solution. For 10^-9^ M concentrations, add 100 mL of stock solution to 900 mL of DDW, For 10^-8^ M concentrations, add 100 mL of 10^-9^ M solution to 900 mL of DDW, for 10^-7^ M concentration add 100 mL of 10^-8^ M solution in 900 mL of DDW, for 10^-6^ M concentration add 100 mL of 10^-7^ M solution in 900 mL of DDW. Control plants were sprayed with DDW only. Karrikinolide was procured from Toronto Research Chemicals, Canada. ISA was procured from Sigma Aldrich, USA. Foliar spray treatments were applied five times with a five-day interval in both experiments. Experimental parameters were recorded after 7 days of the last foliar application.

### Growth biomarkers

Growth parameters, viz. shoot length, FW (fresh weight), DW (dry weight), leaf area, and leaf yield per plant, were determined at 90 DAP (days after plantation). Mint plants were dig up cautiously with undamaged roots and kept under running water to separate all the attached foreign particles; they were surface dried using blotting papers. Shoot lengths were measured using a meter scale. The FW of the whole plant was measured using an analytical balance. Following the measurement of each plant’s FW, the plants were dried in a hot-air oven at 70°C for 24 hours. Later, the dry mass of the plants was measured with the help of a digital balance. Leaf area was calculated using the graph paper method, in which a leaf was placed on graph paper and the area was measured by counting the number of cubes (1 cm^2^) (Watson, 1958). Leaf yield per plant was calculated by weighing all the leaves present per plant with the help of an electronic balance.

### Evaluation of chlorophyll fluorescence (Fv/Fm)

The chlorophyll fluorescence (Fv/Fm) value was measured on the freshly plucked leaf’s surface (adaxial) by using a portable chlorophyll fluorometer (PAM-2000, Walz, Effeltrich, Germany). Chlorophyll fluorescence components, viz., Fv and Fm, represent variable fluorescence and maximum fluorescence, respectively. Chlorophyll fluorescence (Fv/Fm) is also known as the maximum quantum efficiency of PSII photochemistry and was measured by pre-darkening the leaf for 1 second with a PPFD (photosynthetic photon flux density) of 3000 µmol m^−2^ s^−1^ as a saturating flash. As linked parameters, the apparent photosynthetic electron transport rate of PSII activity (ETR) and the effective quantum yield of PSII electron transport [or Y (II)] were also measured. At 1500 μmol m^-2^ s^-1^ actinic light, Y(II) was measured (Nabi et al., 2021).

### Evaluation of total chlorophyll (Chlorophyll a and b) and carotenoid activity

The contents of total chlorophyll and carotenoids were measured using fresh leaves according to the methodology of Lichtenthaler and Buschmann (2001). Freshly extracted tissues from the interveinal leaf area were macerated with acetone (80%) using a clean mortar and pestle. With the help of a spectrophotometer, the OD of the solution was measured at 662, 645, and 480 nm in order to estimate the content of chlorophyll a, chlorophyll b, and total carotenoids, respectively. Total leaf chlorophyll content was calculated by adding the values of chlorophyll a and chlorophyll b. Both chlorophyll and carotenoids were expressed as mg/g^-1^ leaf FW.

### Evaluation of nitrate reductase activity

Nitrate reductase activity was determined using Jaworski (1971) methodology, in which fresh leaves were sliced into small pieces, 200 mg of which were shifted into different plastic vials. Add 2.5 mL of phosphate buffer (pH 7.5) and 0.5 mL of a 0.2 M potassium nitrate (KNO_3_) solution in plastic vials. Thereafter, 2.5 mL of 5% isopropanol was added. To avoid bacterial growth, add two drops of chloramphenicol solution. The plastic vials were incubated in the dark for 2 hours at 30°C using a BOD incubator. The nitrite formed was measured with the help of a spectrophotometer.

### Evaluation of CA (carbonic anhydrase) activity

CA (carbonic anhydrase) activity was measured by the methodology of Dwivedi and Randhawa (1974). 200 mg of freshly chopped leaves were incubated in 10 mL of a 0.2 M aqueous cysteine solution in a Petri dish at 4°C for 20 minutes. Remove the cysteine solution that has adhered to the cut surfaces of the chopped leaves with blotting paper, then transfer them instantly into a test tube having 4 mL of phosphate buffer (pH 6.8). To it, 4 mL of a 0.2 M NaHCO**_3_ (**sodium bicarbonate) solution, 0.02 M of sodium hydroxide (NaOH) solution, and 0.2 mL of a 0.002% bromothymol-blue indicator were added. The liquid content of the solution was titrated against 0.05 N hydrochloric acid using methyl red as an indicator, and the values of the enzyme activity were expressed as µmol CO_2_ kg^-1^ leaf FW s^-1^.

### Evaluation of leaf phenolic content

Sadasivam and Manickam (2008)’s methodology was used to determine the leaf-phenol content. Using a mortar and pestle, 500 mg of dried leaf tissue was macerate with 80% ethanol. Later, the homogenate was transferred into a centrifuge tube, followed by centrifuging the content at 10,000 rpm for 10 minutes at 4°C. After centrifugation, the supernatant was left to dry. It was diluted with 5 mL of DDW. And then, 0.5 mL of Folin-Ciocalteau reagent and 2 mL of a 20% Na_2_CO_3_ solution were added. The optical content of the mixture was recorded at 650 nm by using spectrophotometer against a reagent blank. With the help of the standard curve, the concentration of phenol present in each sample was evaluated and expressed as mg phenol per 100 g^-1^ of dry leaves (mg of phenol per 100 g of leaf powder, DW).

### Evaluation of leaf-flavonoid content

Leaf-flavonoid content was measured according to the methodology of Meda et al. (2005). Regarding this, 5 mL of aluminum trichloride (based on 2% methanol) was mixed with 5 mL of leaf-extract solution, using fresh leaves. After 10 minutes, the absorbance of the sample was measured at 415 nm with the help of a spectrophotometer. To calibrate the spectrophotometer, a blank sample including 5 mL of extract solution homogenized with 5 mL of methanol was used. The leaf-flavonoid content was measured using a standard curve prepared with graded dilutions of catechin (0–100 mg/L). Leaf-flavonoid content was expressed as mg catechin equivalent per 100 g of fresh weight (mg CE 100 mg^-1^ FW).

### Evaluation of essential oil-related attributes

To determine the contents of essential oil (EO) and quality attributes, the contents of menthol and menthyl acetate were measured in the mint EO. The essential oil of *Mentha arvensis* was extracted using the hydro-distillation technique using Clevenger’s apparatus (Borosil, India). 200 g of fresh leaves of *Mentha arvensis* were diced into small pieces prior to EO extraction by the distillation method for 3 hours. The extracted essential oil was dried with anhydrous Na_2_SO_4_ (sodium sulphate) and further preserved at 4°C for GC/MS analysis. The amount of EO obtained from plant material was calculated as follows:


Essential oil (%v/w) = (observed volume of essential oil (mL)/weight of leaves sample (g)) * 100

### SEM study of leaf essential oil glandular trichomes and stomata

A study of leaf glandular trichomes containing essential oil was examined with a scanning electron microscope (SEM) present at the USIF, AMU, Aligarh. For SEM analysis, fresh leaves from each treatment were harvested and sequentially treated with several dilutions of ethanol (30%, 50%, 70%, 90%, and absolute alcohol). Following that, alcohol-dried leaves were coated with gold particles, and the size of glands and stomata was measured in micrometers using a scale bar and SEM. In this experiment, four randomly selected leaves were used from each treatment, and each treatment was replicated five times.

### GCMS analysis of essential oils

Detection of bioactive constituents present in the essential oil of the mint plant was investigated with the help of gas chromatography and mass spectrometry (GC/MS) from AIRF, JNU, and New Delhi. In this experiment, a Perkin Elmer Clarus 680 quadrupole mass spectrometer was attached to a TG-WAX MS-fused silica capillary column (30 meter × 0.25mm ID × 0.25µm df). The injection temperature was 260°C with a split ratio of 1:40. The temperature of the column oven was programmed to go from 50°C to 140°C at a rate of 3°C per minute and a hold of 2 minutes, then from 140°C to 210°C at a rate of 6°C per minute and a hold of 2 minutes, and finally from 210°C to 280°C at a rate of 6°C per minute and a hold of 6 minutes. In addition, helium gas is used as a carrier reagent at a constant flow rate of 1.21 mL/min. The ionization energy of the equipment was 70 eV, and the mass scan range was 40–600 angstrom. At the source, the temperature was 220°C. The characterization of bioactive constituents was realized on the basis of elution order and retention time with the help of the TurboMass NIST 2011 libraries (version 2.3.0) and the Wiley Registry of Mass Spectral Data (9^th^ edition).

### Statistical analysis

All treatments contained five replicates, and a single pot was considered a single replicate. Data were statistically analyzed with SPSS-22 (SPSS Inc., Chicago, IL, USA). DMRT (Duncan multiple range test) was applied at *p ≤* 0.05, and the data means were compared with ± S.E.

## Results

In previous studies, several experiments have been conducted on the application of smoke-saturated water and/or karrikinolide to seed germination, resulting in promising results (Singh et al., 2014; Akeel et al., 2019). Smoke-saturated water is a stable compound that is bioactive at very low concentrations; therefore, it can be used feasibly (Van Staden et al., 2006). In current study, different concentrations of PDSW (1:125, 1:250, 1:500, and 1:1000 v/v) and karrikinolide (10^-9^ M, 10^-8^ M, 10^-7^ M and 10^-6^ M) were applied to the foliage of *Mentha arvensis*. In general, PDSW (1:500 v/v) and KAR_1_ (10^-8^ M) proved the best for growth attributes, physiological parameters, essential oil productivity, and bioactive constituents (menthol, menthone, and menthyl acetate) of *Mentha arvensis*. Detailed results, observed due to the foliar application of PDSW and KAR1, are described below.

### Growth attributes

Growth attributes were determined after 7 days of the last foliar-spray treatment. Different PDSW and KAR1 concentrations stimulated plant growth in terms of shoot length and FW and DW of the shoot. Foliar application of PDSW and KAR1 also increased the leaf number and leaf yield per plant in comparison with the control (DDW). Out of various foliar application treatments, PDSW (1:500 v/v) and KAR_1_ (10^-8^ M) enhanced the shoot length by 19.23% and 16.47%, respectively, in comparison with control. In addition, 1:500 v/v of PDSW enhanced the FW and DW of the plant by 19.30% and 35.36%, respectively, while 10^-8^ M of KAR_1_ proved the best KAR_1_ treatment in this regard, increasing the FW and DW of the plant by 17.44% and 24.75%, respectively, in comparison with control. Similarly, PDSW (1:500 v/v) and KAR_1_ (10^-8^ M) increased the leaf number by 28.41% and 23.74%, and the leaf area by 18.22% and 17.46%, respectively, in comparison with control (**Table 1**).

**Table 1:** Effect of foliar application with PDSW and KAR1 on shoot height, fresh weight, dry weight, leaf number and leaf area per plant of *Mentha arvensis* L. recorded at 90 DAP (days after plantation).

Treatments	Shoot length (cm)	Fresh weight (g plant^-1^)	Dry weight (g plant^-1^)	Leaf number plant^-1^	Leaf area plant^-1^ (cm^2^)
**PDSW Control**	64.1±0.37^e^	59.2±0.46^e^	8.2±0.20^c^	85.66±1.48^d^	2639±11.26^e^
**1:125 v/v**	68.2±0.43^d^	63.5±0.34^d^	8.7±0.26^c^	92.66±1.45^c^	2810±6.50^d^
**1:250 v/v**	70.06±0.58^c^	65.06±0.63^c^	9.46±0.24^b^	100.33±1.45^b^	2990±17.63^c^
**1:500 v/v**	76.43±0.53^a^	70.63±0.37^a^	11.1±0.25^a^	110±2.64^a^	3120±30.55^a^
**1:1000 v/v**	73.43±0.31^b^	68.36±0.42^b^	10.63±0.24^b^	105±2.08^b^	3010±6.35^b^
**KAR_1_ Control**	64.1±0.37^e^	59.2±0.46^e^	8.2±0.20^d^	85.66±1.48^d^	2639±11.26^d^
**10^-9^ M**	71.73±0.32^b^	67.13±0.54^b^	9.98±0.07^b^	100±0.57^b^	2915±31.13^b^
**10^-8^ M**	74.66±1.03^a^	69.53±0.17^a^	10.23±0.20^a^	106±1.15^a^	3100±21.51^a^
**10^-7^ M**	69.73±0.32^c^	65.26±0.56^c^	8.43±0.32^b^	94.66±1.45^b^	2871±8.81^b^
**10^-6^ M**	66.2±0.57^d^	62.53±0.49^d^	7.53±0.20^c^	90.12±1.52^c^	2715±11^c^

Each value, shown with ± SE, represents the mean of 6 replicates. Means within a column, followed by the same letter(s), are not significantly different (p ≤ 0.05) according to DMRT. PDSW- plant derived smoke water, Control -DDW (double distilled water), KAR_1_- karrikinolide.

### Photosynthetic yield

Foliar application of PDSW and karrikinolide enhanced the photosynthetic yield (effective quantum yield of photosystem II and chlorophyll and carotenoid yield) of the mint plant. PDSW (1:500 v/v) and KAR_1_ (10^-8^ M) enhanced the chlorophyll fluorescence (Fv/Fm) values by 12.10% and 11.41%, respectively, in comparison with control. In addition, 1:500 v/v of PDSW increased the qP (photochemical quenching), qN (non-photochemical quenching), Y (II) (effective quantum yield of photosystem II), and ETR (electron transport rate) values by 26.93%, 41.11%, 33.91%, and 22.12%, respectively, in comparison with the control treatment (DDW-spray). Similarly, foliar application of KAR_1_ (10^-8^ M) significantly increased the values of qP, qN, Y (II), and ETR by 25.74%, 35.55%, 33.52%, and 13.74%, respectively (**Table 2**).

**Table 2:** Effect of foliar application with PDSW and KAR**_1_** on chlorophyll fluorescence (Fv/Fm) and the related parameters (qP, qN, Y(II), ETR) measured on the leaves of *Mentha arvensis* L. at 90 DAP (days after plantation).

Treatments	Chlorophyll fluorescence (Fv/Fm)	qP	qN	Y(II)	ETR
**PDSW Control**	0.727±0.0017^e^	0.672±0.003^e^	0.090±0.005^e^	0.516±0.004^e^	25.9±0.23^d^
**1:125 v/v**	0.763±0.0025^d^	0.721±0.006^d^	0.094±0.002^d^	0.564±0.004^d^	26.16±1.09^c^
**1:250 v/v**	0.799±0.002^c^	0.816±0.004^c^	0.100±0.005^c^	0.615±0.003^c^	27.63±0.61^b^
**1:500 v/v**	0.815±0.004^a^	0.853±0.002^a^	0.127±0.004^a^	0.691±0.003^a^	31.63±0.24^a^
**1:1000 v/v**	0.801±0.002^b^	0.840±0.005^b^	0.110±0.002^b^	0.662±0.004^b^	29.2±0.416^b^
**KAR_1_ Control**	0.727±0.0017^e^	0.672±0.003^d^	0.090±0.005^c^	0.516±0.004^e^	25.9±0.23^d^
**10^-9^ M**	0.799±0.009^b^	0.832±0.002^b^	0.111±0.002^b^	0.662±0.004^b^	28.66±0.62^ab^
**10^-8^ M**	0.810±0.004^a^	0.845±0.003^a^	0.122±0.004^a^	0.689±0.005^a^	29.46±1.27^a^
**10^-7^ M**	0.765±0.003^c^	0.810±0.003^b^	0.101±0.006^b^	0.621±0.002^c^	27.1±0.61^bc^
**10^-6^ M**	0.748±0.003^d^	0.741±0.006^c^	0.096±0.002^c^	0.555±0.004^d^	26.96±0.44^cd^

Each value, shown with ± SE, represents the mean of 6 replicates. Means within a column, followed by the same letter(s), are not significantly different (p ≤ 0.05) according to DMRT. Control - DDW (double distilled water), PDSW, plant-derived smoke-water; KAR**_1_**, karrikinolide; qP, photochemical quenching; qN, non-photochemical quenching; Fv, variable fluorescence; Fm, maximum fluorescence; Y(II), effective quantum yield of photosystem II, ETR; electron transport rate.

**Figure 1:**
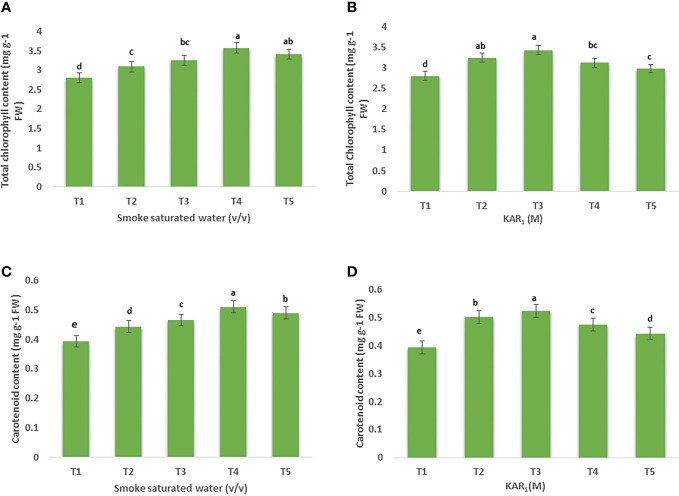
Effect of foliar application with smoke-saturated water 1:125, 1:250, 1:500, and 1:1000 v/v) and karrikinolide (10^-9^ M, 10^-8^ M, 10^-7^ M and 10^-6^ M) on total chlorophyll content (mg g^-1^) **(A, B)** total carotenoid content (mg g^-1^) **(C, D)** of *Mentha arvensis* L. Lowercase letters depicts the Duncan. Data are presented as treatments mean ± SE (n = 5). Data followed by the same letters are not significantly different by Duncan multiple range test at *p* ≤ 0.05.

Foliar application of 1:500 v/v of PDSW enhanced the total chlorophyll content of mint by 25.70% in comparison with control (**Figure 1A**). Similarly, treating the mint plant with KAR_1_ (10^-8^ M) significantly enhanced the values of total chlorophyll content by 20.77% (**Figure 1B**). In addition, the leaf-carotenoid content of mint was also enhanced when treated with 1:500 v/v of PDSW and 10^-8^ M of KAR_1_ by 29.77% and 27.18%, respectively, in comparison with control treatment (**Figures 1C, D**).

### CA and NRA activity

**Figure 2:**
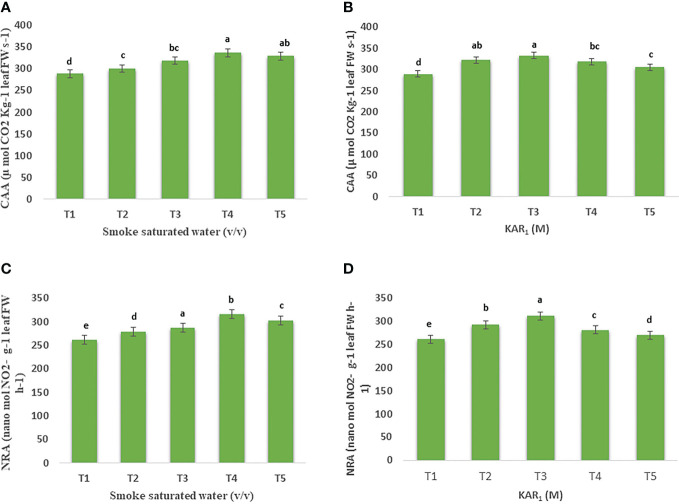
Effect of foliar application with smoke-saturated water 1:125, 1:250, 1:500, and 1:1000 v/v) and karrikinolide (10^-9^ M, 10^-8^ M, 10^-7^ M and 10^-6^ M) on carbonic anhydrase activity (µmol CO_2_ Kg^-1^ leaf FW s^-1^) **(A, B)** and nitrate reductase activity (nanomol NO_2_^-^ g^-1^ leaf FW h^-1^) **(C, D)** of *Mentha arvensis* L. Lowercase letters depicts the Duncan. Data are presented as treatments mean ± SE (n = 5). Data followed by the same letters are not significantly different by Duncan multiple range test at *p* ≤ 0.05.

Foliar application of PDSW (1:500 v/v) and KAR_1_ (10^-8^ M) also enhanced the enzyme activities maximally in comparison with the control. Foliar feeding with PDSW (1:500 v/v) and KAR_1_ (10^-8^ M) increased the activity of carbonic anhydrase (CA) by 16.56% and 15.13%, respectively (**Figures 2A, B**). Likewise, PDSW (1:500 v/v) and KAR_1_ (10^-8^ M) increased the activity of nitrate reductase (NR) by 20.89% and 18.33%, respectively (**Figures 2C, D**).

### Contents of phenols and flavonoids

**Figure 3:**
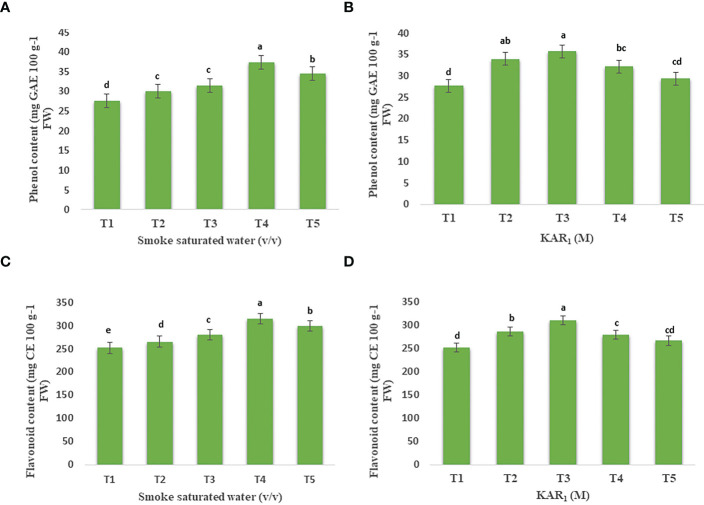
Effect of foliar application with smoke-saturated water 1:125, 1:250, 1:500, and 1:1000 v/v) and karrikinolide (10^-9^ M, 10^-8^ M, 10^-7^ M and 10^-6^ M)on phenol content (mg GAE 100 g^-1^ FW) **(A, B)** and flavonoid content (mg CE 100 g^-1^ FW) **(C, D)** of *Mentha arvensis* L. Lowercase letters depicts the Duncan. Data are presented as treatments mean ± SE (n = 5). Data followed by the same letters are not significantly different by Duncan multiple range test at *p* ≤ 0.05.

1:500 v/v of PDSW and 10^-8^ M of KAR_1_, applied to mint foliage, maximally enhanced the contents of phenols and flavonoids in comparison with the control. Foliage-applied PDSW (1:500 v/v) and KAR_1_ (10^-8^ M) increased the phenol content by 34.99% and 29.38%, respectively (**Figures 3A, B**). Similarly, 1:500 v/v of PDSW and 10^-8^ M of KAR_1_ increased the leaf-flavonoid content by 25.06% and 23.39%, respectively (**Figures 3C, D**).

### SEM analysis

**Figure 4:**
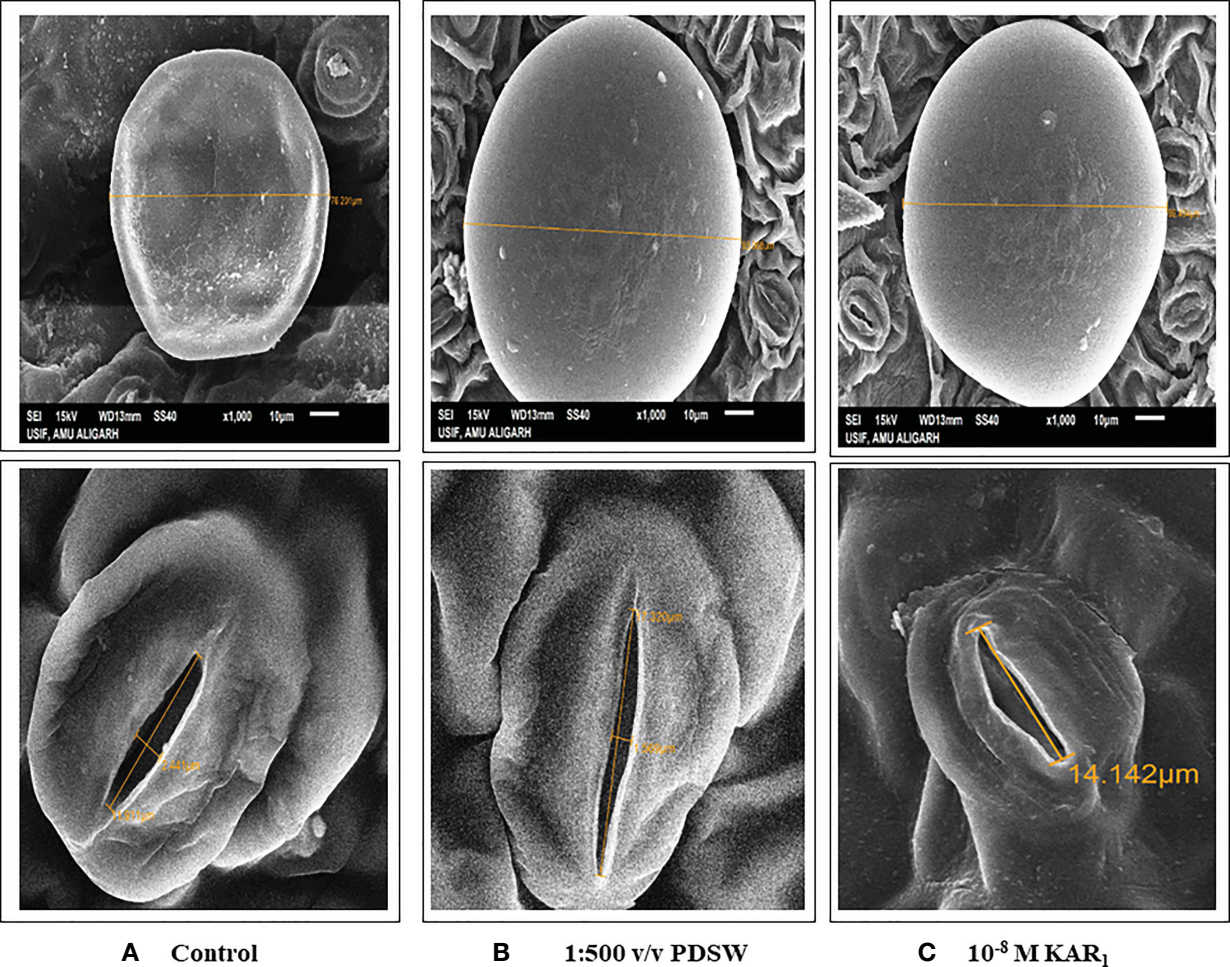
Response of *Mentha arvensis* L. to foliar treatment with smoke-saturated water (PDSW) and KAR**_1_** regarding size of leaf glandular trichomes and length of stomatal pore as observed under scanning electron microscope as affected by **(A)** Control **(B)** 1:500 v/v of PDSW and **(C)** 10^-8^ M of KAR_1_.

Foliage applied with PDSW (1:500 v/v) and KAR_1_ (10^-8^ M) enhanced the size of glandular trichomes and stomatal pores maximally, in comparison with control treatment. 1:500 v/v of PDSW enhanced the size of glandular trichomes by 24.75%, while 10^-8^ M of KAR_1_ increased the glandular-trichome size by 17.32% in comparison with the control. Moreover, foliar application of 1:500 v/v of PDSW increased the pore length of stomata by 45.42%, while 10^-8^ M of KAR_1_ increased it by 18.72%, respectively, in comparison with control treatment (**Figure 4**).

### Content and productivity of essential oils and bioactive constituents

**Figure 5:**
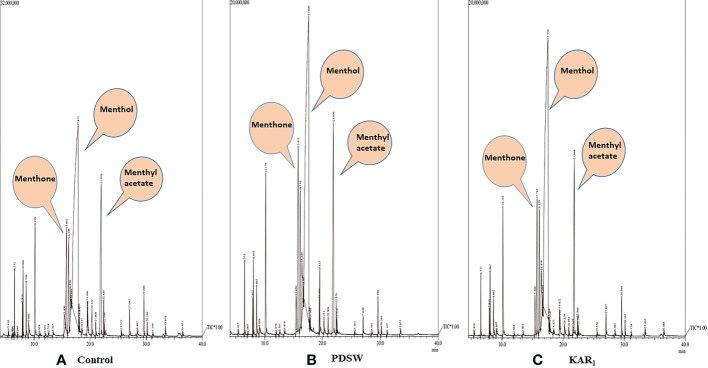
GC-MS chromatogram of essential oil of *Mentha arvensis* L. **(A)** Control, **(B)** 1:500 v/v of PDSW and **(C)** 10^-8^ M of KAR**_1_**.

**Figure 6:**
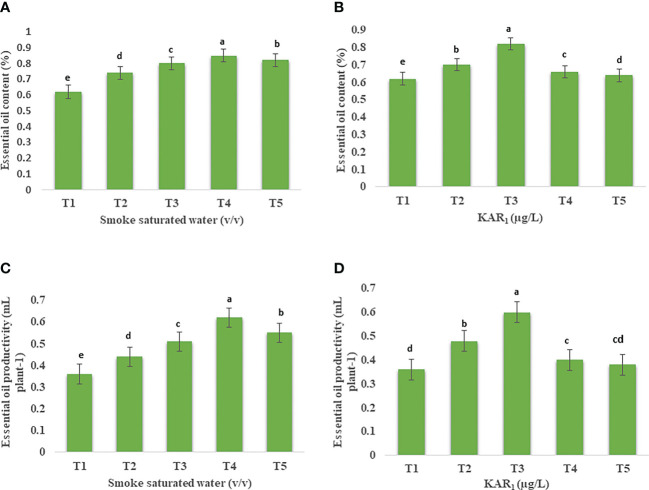
Effect of foliar application with **(A)** smoke-saturated water (32.25 µg/L, 64.5 µg/L, 1: 500 v/vand 258 µg/L) and KAR_1_ (0.150 µg/L, 1.501 µg/L, 15.013 µg/L and 150.13 µg/L) on essential oil content (%) **(A, B)** and essential oil productivity (mL plant^-1^) **(C, D)** of *Mentha arvensis* L. Lowercase letters depicts the Duncan. Data are presented as treatments mean ± SE (n = 5). Data followed by the same letters are not significantly different by Duncan multiple range test at *p* ≤ 0.05.

Out of various foliar-spray concentrations, PDSW (1:500 v/v) and KAR_1_ (10^-8^ M) proved the best, increasing the content as well as productivity of mint essential oil (EO) maximally in comparison with control (**Figures 5**, **6**). GCMS analysis revealed that PDSW (1:500 v/v) and KAR_1_ (10^-8^ M) increased the EO content by 37.09% and 32.25%, respectively (**Figures 6A, B**), while application of PDSW (1:500 v/v) and KAR_1_ (10^-8^ M) increased the EO productivity of mint by 72.22% and 66.66%, respectively (**Figures 6C, D**).

According to the GCMS report, foliar applications of 1:500 v/v of PDSW and 10^-8^ M of KAR_1_ also increased the percent area of bioactive constituents as detected by the total ion chromatogram (**Table 3**). The following bioactive constituents were found in the essential oil of the mint plant: menthol, menthyl acetate, menthone, limonene, cyclohexanone, 5-methyl-2-(1-methylethenyl)-, (2R-cis)-, α-pinene, β-pinene, myrcene, cyclohexanol, 5-methyl-2-1-me htylethenyl)-, neomenthol, piperitone, germacrene D (**Table 3**). In comparison with control, PDSW (1:500 v/v) and KAR_1_ (10^-8^ M) increased the menthol content by 29.94% and 25.42%, respectively. In addition, foliar application of PDSW (1:500 v/v) and KAR_1_ (10^-8^ M) increased the menthyl acetate content by 36.90% and 31.73% and the menthone content by 44.38% and 37.75%, respectively, in comparison with control treatments (**Table 3**).

**Table 3:** Effect of foliar application with PDSW and KAR**_1_** on TIC (Total ion chromatogram) of the essential oil composition measured by GCMS on the leaves of *Mentha arvensis* L. at 90 DAP (days after plantation).

S. No.	Compound detected	Retention time range (RTR)	Control (% Area)	PDSW (1:500 v/v) (% Area)	KAR_1_ (10^-8^ M) (% Area)
1.	α-Pinene	6.531-6.532	0.85	1.01	0.90
2	β-Pinene	8.062-8.066	1.01	1.19	1.14
3	Myrcene	8.598-8.603	0.96	1.22	1.10
4	Limonene	10.163-10.190	1.09	0.46	1.18
5	Cyclohexanol, 5-methyl-2-(1-menthylethenyl)-	15.400-15.450	0.51	0.82	0.70
6	Menthone	15.743-15.811	3.47	5.01	4.78
7	Cyclohexanone, 5-methyl-2-(1-menthylethenyl)-,(2R-cis)-	16.129-16.188	1.70	0.85	1.02
8	Neomenthol	16.439-16.450	0.83	0.98	0.85
9	Menthol	17.539-17.871	60.72	78.90	76.16
10	Piperitone	20.228-20.322	0.46	0.51	0.48
11	Menthyl acetate	21.896-22.360	5.23	7.16	6.89
12	Germacrene D	29.589-29.606	0.81	0.95	0.99

PDSW, plant-derived smoke-water; KAR_1_, karrikinolide.

## Discussion

Medicinal plants have become an interesting subject of research as it pertains to the industrial and pharmacological sectors. The essential oil of *Mentha arvensis* contains several bioactive constituents like menthol, menthyl acetate, isomenthone, etc. that have pharmacological values. PDSW and its bioactive constituents, including KAR1, have previously been reported as promising germination stimulants (Singh et al., 2014; Akeel et al., 2019). The current study reveals the effects of foliar application of PDSW and karrikinolide (KAR_1_) on growth, physiological parameters, and essential oil production in the mint plant. Foliar application, in general, reduces the time lag between soil application and plant uptake because it is fed directly to leaves (Taiz et al., 2014). According to Kulkarni et al. (2007), foliar application of smoke-saturated water (1:500 v/v) and smoke-isolated butenolide resulted in enhancement of growth parameters including shoot and root length, fresh and dry weight, leaf number, leaf area, and thickness of the stem, in addition to improving the absolute growth rate and seedling vigor index of okra and tomato seedlings. As per another experiment conducted by Kulkarni et al. (2008) under greenhouse conditions, application of both smoke-saturated water and smoke-isolated butenolide improved the plant height, leaf number, and stem thickness of tomato plants in comparison with the control. In current study, foliar application of plant-derived smoke-saturated water (PDSW) and karrikinolide (KAR**_1_**) on mint plants resulted in promising outcomes. Of the foliar-spray concentrations used as PDSW (1:125, 1:250, 1:500, and 1:1000 v/v) and KAR**_1_** (10^-9^ M, 10^-8^ M, 10^-7^ M and 10^-6^ M), PDSW (1:500 v/v) and KAR_1_ (10^-8^ M) gave the best results for all of the growth parameters studied (shoot length, fresh weight and dry weight of the plant, leaf area, and leaf yield per plant). Similarly, smoke-saturated water improved the growth-related parameters of papaya seedlings, viz. shoot (50.45%), shoot fresh and dry weight (96.59% and 88.88%), and leaf number (14.94%), in comparison with the control (Chumpookam et al., 2012). Likewise, 1:500 v/v of PDSW exhibited similar results in the current experiment *via* improving the growth-related parameters, including plant height (19.23%), fresh weight (19.30%), dry weight (35.36%), leaf area (18.22%), and leaf yield per plant (28.41%). Here, both PDSW and karrikinolide showed growth-promoting activities similar to gibberellic acid (Singh et al., 2014). Likewise, smoke-saturated water and karrikinolide increased the seedling fresh weight and seedling axis of *Phaseolus vulgaris* L. (Singh et al., 2014). In the current experiment, 10^-8^ M of KAR_1_ also showed similar results, increasing the plant height (16.47%), fresh weight (17.44%), dry weight (24.75%), leaf area (17.46%), and leaf yield per plant (23.74%), in comparison with the control. The above discussion suggested that both PDSW and karrikinolide might be used as foliar-application treatments to improve the growth of mint plants or other crops.

In view of the current study, PDSW (1:500 v/v) and KAR_1_ (10^-8^ M) showed significantly enhanced results regarding chlorophyll-fluorescence values in comparison with control treatment. According to Zhou et al. (2013), smoke-saturated water enhanced the chlorophyll fluorescence values, PS (II) activity, and photochemical quenching of *Isatis indigotica* seedlings in comparison with control. In another study conducted on carrot (*Daucus carota* L.), smoke-saturated water and karrikinolide improved the values of chlorophyll fluorescence as compared to the control (Akeel et al., 2019). These findings suggested that treatments (1:500 v/v of PDSW and 10^-8^ M of KAR_1_) applied as foliar sprays on mint plants in the present study might improve the stomatal opening, as supported by the SEM analysis of the leaf (stomatal pore size), which, in turn, facilitated improved values of photosynthesis-related parameters (Fv/Fm, qP, qN, Y(II), and ETR).

Photosynthetic pigments are the regulators of photosynthesis and, hence, are important for plant growth as a whole (Parida and Das, 2005). Photosynthetic pigments, including chlorophyll a, chlorophyll b, and carotenoids, are essential for photosynthesis as well as the growth and development of plants (Bollivar, 2006). Treatments PDSW (1:500 v/v) and KAR_1_ (10^-8^ M) increased the total leaf content of chlorophyll and carotenoids in comparison with the control. According to Aslam et al. (2017), 1: 500 v/v of smoke-saturated water increased the total content of chlorophyll and carotenoids in maize seedlings. As per a recent finding, application of KAR_1_ (10^-6^ M) increased the total chlorophyll and carotenoid contents of *Coriandrum sativum* under both stressful and non-stressful conditions (Sardar et al., 2021).

In the current study, foliar application of both PDSW and its bioactive constituent KAR**_1_** increased the activity of the nitrogen-assimilation-related enzyme nitrate reductase (NR). Treatments with 1: 500 v/v of PDSW and 10^-8^ M of KAR_1_ exhibited significant results, expressing enhancements in the values of NR activity by 20.89% and 18.33%, respectively, in comparison with the control. A proteomic analysis revealed that plant-derived smoke water promotes chickpea growth while also increasing nitrate metabolism-related proteins (Rehman et al., 2018).This might be possible since plant-derived smoke water and karrikinolide help increase the NR activity, which might indicate an associated increase in the nitrate content of plants. Carbonic anhydrase (CA) is another important enzyme that catalyzes the interconversion between carbon dioxide and bicarbonate (Supuran, 2016). CA plays a significant role in providing carbon dioxide to RuBisCo during the process of photosynthesis (Taiz et al., 2014). In the present investigation, foliar application of PDSW and karrikinolide increased the CA activity values in comparison with the control (34.05% and 27.42%).

Phenols and flavonoids, the secondary metabolites in plants, are good electron donors, due to which they contribute to antioxidant action under stressful conditions (Bendary et al., 2013). In the current study, the treatments PDSW (1:500 v/v) and KAR1 (10^-8^ M) significantly increased the phenols and flavonoids content.In this context, a transcriptomic analysis revealed that plant-derived smoke water and karrikinolide increased the expression of genes related to flavonoids and phenylpropanoid pathways (Soós et al., 2010). Aremu et al. (2012) investigated the positive effect of smoke-saturated water and karrikinolide on the accumulation of phenols and flavonoids in developing plantlets of “Williams” bananas. A metabolomics study, conducted by Sun et al. (2020), revealed the occurrence of 178–199 differential metabolites in *Salvia miltiorrhiza* plants when treated with smoke water and karrikinolide. These metabolites were assigned to different metabolic pathways, including the TCA cycle, glycolytic pathways, the biosynthesis of flavonoids and terpenoids, etc. Hence, PDSW and karrikinolide, applied as foliar sprays on mint plants in this study, might have stimulated the expression of genes related to phenol and flavonoid pathways, resulting in the significant enhancement of phenols and flavonoids in mint plants.

In the present investigation, foliar application of PDSW and KAR_1_ increased the stomatal pore size as well as the content and productivity of essential oil (EO) of *Mentha arvensis* L. In support of the present findings, Akeel et al. (2019) reported a significant increase in stomatal pore length in carrot leaves as a result of application of smoke-saturated water and karrikinolide in comparison with control. Similar findings regarding a significant increase in the stomatal pore size of mint leaves were revealed in the current study due to foliar application of PDSW and karrikinolide Naeem et al. (2012) discovered a similar effect of irradiated sodium alginate (ISA), reporting significant improvements in the content and yield of essential oil of *Mentha arvensis* L. after foliar application of ISA, a growth promoter. Similar findings were also reported in the current experiment, in which foliar application of 1:500 v/v of PDSW and 10^-8^ M of KAR_1_ proved highly fruitful for increasing the EO content and EO productivity of the mint plant. These treatments also significantly improved the contents of the bioactive constituents of mint oil, viz., menthol, menthyl acetate, and menthone. A similar study, conducted by Bose et al. (2013), revealed that the growth promotor gibberellic acid and calliterpenone increased the density of glandular trichomes on mint leaves, which was accordingly manifested in a significantly higher EO yield and bioactive constituents in mint EO. The above-mentioned results might support our investigation, which revealed that PDSW and karrikinolide increased the density of glandular trichomes, facilitating significantly higher EO production in mint.

**Figure 7:**
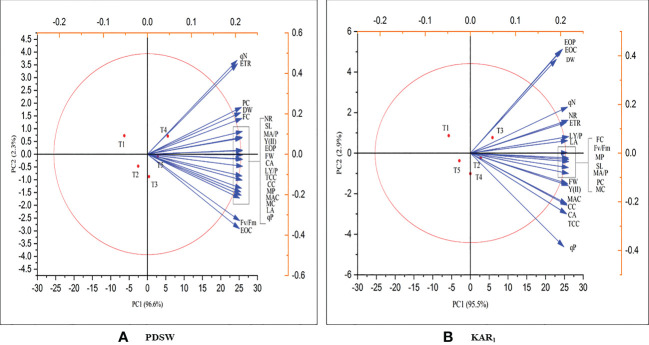
Principal correlation analysis (PCA) shows the positive correlation of the response of **(A)** PDSW and **(B)** KAR_1_ in terms of growth, physiological attributes, essential oil yield and active constituents of mint plant. Foliar application with PDSW and KAR_1_ positively enhances SL, shoot length; FW, fresh weight; DW, dry weight; LA, leaf area; LY/P, leaf yield per plant, NR, nitrate reductase; CA, carbonic anhydrase; PC, phenol content; FC, flavonoid content; EOC, essential oil content; EOP, essential oil production, MC, menthol content; MP, menthol productivity; MAC, menthyl acetate content; MAP, menthyl acetate productivity; TCC, total chlorophyll content; CC, carotenoid content; Fv/Fm, chlorophyll fluorescence; qP, photochemical quenching; qN, (non-photochemical quenching); Y (II), effective quantum yield of photosystem II; and ETR, electron transport rate.

Principal component analysis is a technique that is used to identify the relationship between traits, their variation and PDSW and KAR_1_ priming can improve the growth, physiological attributes and essential oil yield of mint plant. Both of the treatment, PDSW and KAR_1_ significantly increases the plant growth, photosynthetic parameters, essential oil yield and active constituents of essential oil of mint plant as compared to control and they were positively correlated. In PDSW treated plants, PC1 and PC2 contribute variation of 96.6% and 2.3% (**Figure 7A**). Likewise, in KAR_1_ treated plants, PC1 and PC2 contribute to 95.5% and 2.9% (**Figure 7B**). Both treatments (PDSW and KAR_1_) shows significant contribution in enhancing the parameters regarding growth, photosynthesis, physiological and essential oil yield and active constituents.

### Possible perception of plant-derived smoke water or karrikinolide by the mint plant

**Figure 8:**
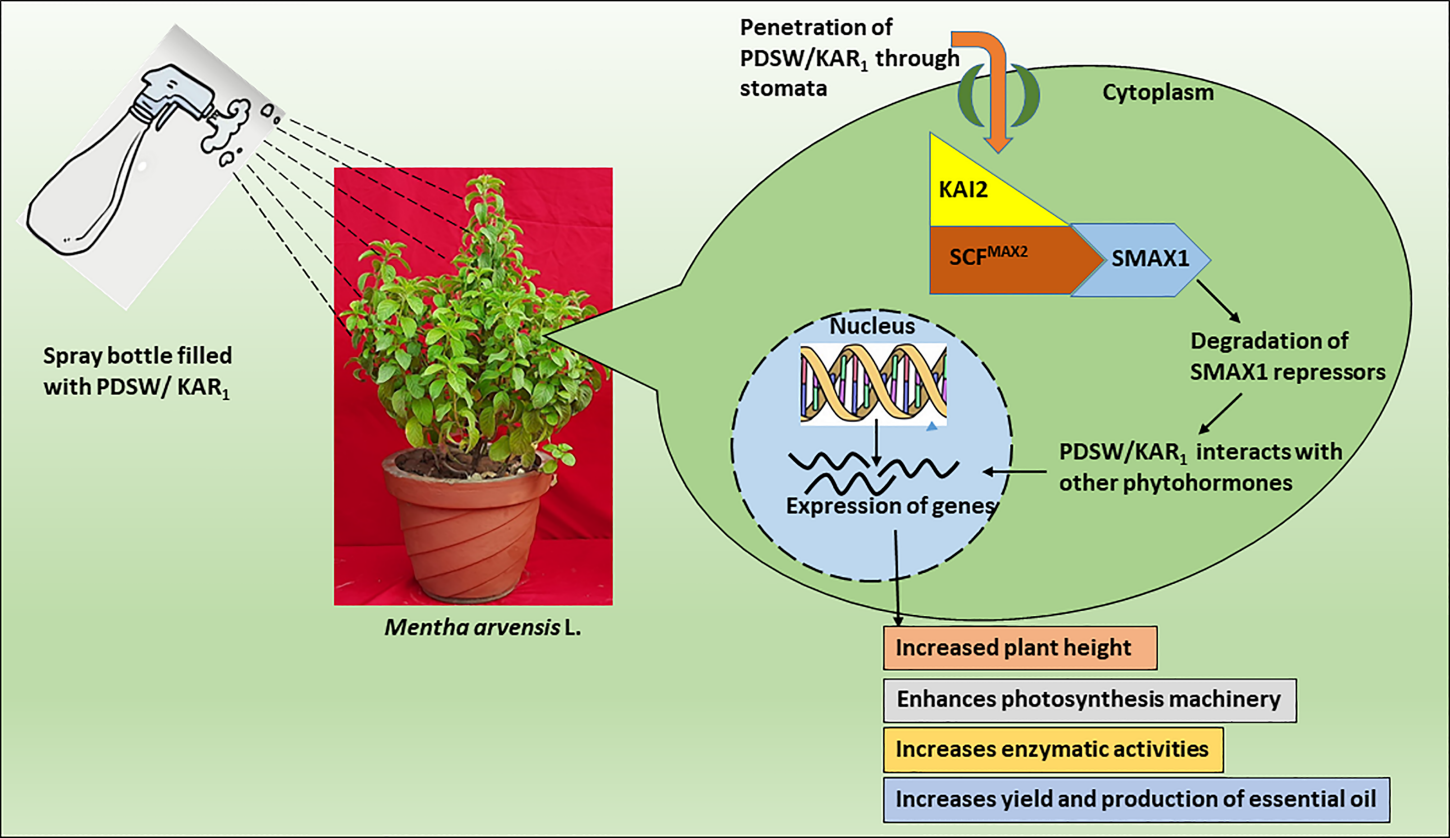
Possible schematic representation of perception by mint plant and mechanism of action of PDSW/KAR_1_. PDSW/KAR_1_ is absorbed through stomata of mint-plant leaves. PDSW/KAR_1_ is perceived by KAI2 protein that forms a proteosomal complex with MAX2 protein and SMAX1 repressors. After ubiquitin-mediated proteosomal degradation of repressors, PDSW/KAR_1_ interacts with indigenous phytohormones that enhance the expression of genes related to growth, development and essential oil productivity of mint plant.

A number of studies have been conducted so far to reveal the mechanism of action related to plant-derived smoke water or karrikins, but the complete process is still a mystery. Several studies have revealed similarities between the action mechanisms of smoke-saturated water and karrikinolide and those of plant growth regulators associated with the growth and development of plants (Singh et al., 2014; Kepczyński, 2020). Smoke-saturated water/karrikinolide plays a potent role in the seed germination process, similar to gibberellic acid (Drewes et al., 1995; Van Staden et al., 1995). Smoke-saturated water and karrikinolide were directly fed to the leaves of the mint plant, as were similar nutrients, growth regulators, or elicitors, as it might decrease the lag time between soil application and plant uptake (Taiz et al., 2014). In this study, foliage-applied PDSW and karrikinolide on mint plants might get directly absorbed through the stomata of leaves, followed by their penetration into plant cells and transport through apoplastic or symplastic pathways to different parts of the plant (Hong et al., 2021). Thereafter, PDSW or KAR_1_ might interact with two genes, *KAI2* (*KARRIKIN INSENSITIVE2*) and *MAX2* (*MORE AXILLARY GROWTH2)*, that could play a crucial role in the action and signaling mechanisms of karrikin (Smith and Li, 2014; Waters et al., 2014). According to **Figure 8**, the product of KAI2 (KARRIKIN-INSENSITIVE2), a member of the/hydrolase family, may function as both a karrikin receptor and an enzyme in response to signaling mechanisms (Guo et al., 2013). After perceiving PDSW/karrikin, the product KAI2 might form a complex with the MAX2 protein; it later might form the SCF complex (a multi-protein E3 ubiquitin ligase complex), which might help in the degradation of repressors like SMAX1 and SMAX2 (Stirnberg et al., 2002; Nelson et al., 2011). In the process of degradation, the SCF complex might ligate with the ubiquitin bodies, degrading the target proteins *via* 26 proteasomal complexes (Stirnberg et al., 2007). Receptor-protein KAI2 possesses a catalytic triad of amino acid residues (Ser95-His246-Asp217), in which amino acid Ser95 is responsible for karrikin action *via* a nucleophilic attack of Ser95 on the butenolide ring of karrikin (Scaffidi et al., 2012; Waters et al., 2014). Concomitantly, smoke-saturated water or karrikinolide might interact with indigenous phytohormones to execute the plant responses regarding growth and development (Akeel et al., 2019). Such interactions might support improvements in the morphological, physiological, and yield-related parameters of the mint plant.

## Conclusion

In the current study, smoke saturated water, and karrikinolide was applied to the foliage of the mint plant to increase its growth, productivity, and quality. Out of various concentrations of PDSW and karrikinolide used as foliar-spray treatments, PDSW (1:500 v/v) and KAR_1_ (10^-8^ M) clearly exhibited a positive effect on growth, productivity, and quality of the mint plant, suggesting the beneficial use of smoke technology on plants propagating vegetatively. In summary, application of PDSW might be used as a low-cost and sustainable option in improving yield and essential oil production of mint plant. A representation of current work that how application of PDSW and KAR_1_ enhances the morphological growth, physiological attributes and essential oil yield of mint plant is presented in graphical abstract. However, more research should be done to determine the use of foliar application of smoke water on field crops in order to ensure promising results in terms of growth productivity and quality.

## Data availability statement

The raw data supporting the conclusions of this article will be made available by the authors, without undue reservation.

## Author contributions

SarS drafted the original manuscript. AC, SanS and UB prepared the draft of Tables. MU and MK framed and edited the final version of the manuscript figures and tables. All authors contributed to the article and approved the submitted version.
